# Supplementary material for: DnaJC7 binds natively folded structural elements in tau to inhibit amyloid formation
Source: Nat Commun. 2021 Sep 9;12:5338. doi: 10.1038/s41467-021-25635-y (PMC8429438; doi:10.1038/s41467-021-25635-y)
Supplement: Supplementary file 1 — Supplementary Information file [file 41467_2021_25635_MOESM1_ESM.pdf]

SUPPLEMENTARY INFORMATION

DnaJC7 binds natively folded structural elements in tau to inhibit amyloid formation

Zhiqiang Hou, Pawel M. Wydorski, Valerie A. Perez, Aydé Mendoza-Oliva, Bryan D. Ryder, Omar Kashmer, Hilda Mirbaha, Lukasz A. Joachimiak

SUPPLEMENTARY TABLES

Target Gene Symbol	Genomic Sequence	Strand	sgRNA Target Sequence	Target Context Sequence
DNAJC7	NC_000017.11	sense	AAATGCTCAGGCACAACAAG	ATAAAAATGCTCAGGCACAACAAGAGGTAC
DNAJC7	NC_000017.11	antisense	CCTTCCAAGCATCATCAAGG	GGAACCTTCCAAGCATCATCAAGGTGGCTG
DNAJC7	NC_000017.11	sense	GCAAGTGCCACCTCTCTCTG	GAGGGCAAGTGCCACCTCTCTCTGGGGAAT
DNAJC7	NC_000017.11	Sense	GCTAAACTCTACTGTAATCG	AAATGCTAAACTCTACTGTAATCGGGGTAC

Supplementary Table 1. CRISPR gRNA sequences used to knock-out DnaJC7.

SUPPLEMENTARY FIGURES

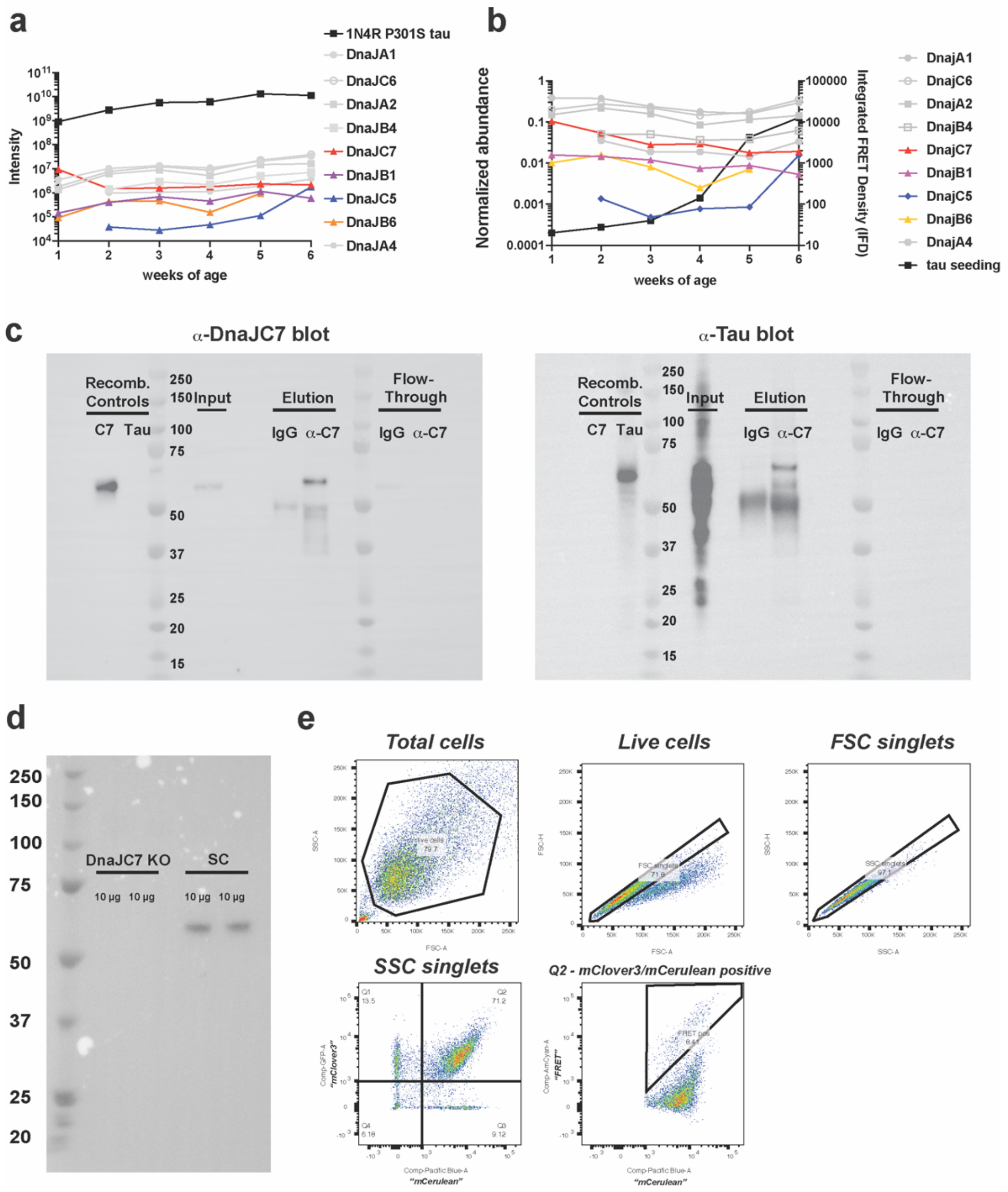

Supplementary Figure 1. Co-Immunoprecipitation of tau and associated JDP chaperones. **a**. Mass spectrometry analysis of tau immunoprecipitation from PS19 tauopathy mice across different ages (weeks 1 through 6) reveals increasing levels of tau (black squares) and identified 9 JDPs associated with tau in each sample. DnaJA1 (grey closed circles), DnaJC6 (grey open circles), DnaJA2 (grey closed squares), DnaJB4 (grey open squares) and DnaJA4 (grey closed hexagons) tracked with tau levels. DnaJB6 (closed orange triangles) and DnaJB1 (closed magenta triangles) levels increased but absolute levels were low. DnaJC7 (closed red triangles) abundance decreased while DnaJC5 (closed blue diamonds) abundance increased. The mass spectrometry data were analyzed using Proteome Discoverer (Thermo). Missing points indicate no detectible signal for that JDP was observed (DnaJB4, DnaJC5, DnaJB6 and DnaJA4). **b.** The immunoprecipitated tau from PS19 tauopathy mice across different ages (weeks 1 through 6)

reveals presence of seeds at early ages (black closed squares). The levels of JDPs were normalized to the signal intensity of tau in each sample. DnaJA1 (grey closed circles), DnaJC6 (grey open circles), DnaJA2 (grey closed squares), DnaJB4 (grey open squares) and DnaJA4 (grey closed hexagons) remained flat, while DnaJB6 (closed orange triangles), DnaJB1 (closed magenta triangles) decreased modestly while DnaJC7 (closed red triangles) decreased. DnaJC5 (closed blue diamonds) abundance increased. Isolated tau from the different samples were evaluated for the presence of pathogenic seeds using tau biosensors (right y-axis) and the normalized abundance of DnaJC7 (relative to P301S 1N4R tau) was determined using mass spectrometry (left y-axis). Seeding experiments were performed as biological triplicates. The mass spectrometry data were analyzed using Proteome Discoverer (Thermo). **c**. Western blots of immunoprecipitations of DnaJC7 from PS19 mouse brains were probed with antibodies against DnaJC7 (left) and tau (right). Each blot contains recombinant proteins (DnaJC7 and 2N4R tau) as well as inputs (2%), flowthroughs, and elutions for IgG control and Anti-DnaJC7 immunoprecipitations. Elution fractions contain background signal for anti-bodies used in the immunoprecipitation (at ~50kDa) despite using specialized secondary antibodies to reduce cross reactivity. IP and western was carried out 3 independent times. **d**. Western blots of HEK293T tau biosensor cell lines treated with gRNAs to create KO DnaJC7 and scrambled non-targeting control cell lines (SC) used in this study. IP and western was carried out 3 independent times. **e**. Gating strategy to measure the fraction of HEK293 tau biosensor cells that contain tau aggregates and are FRET positive.

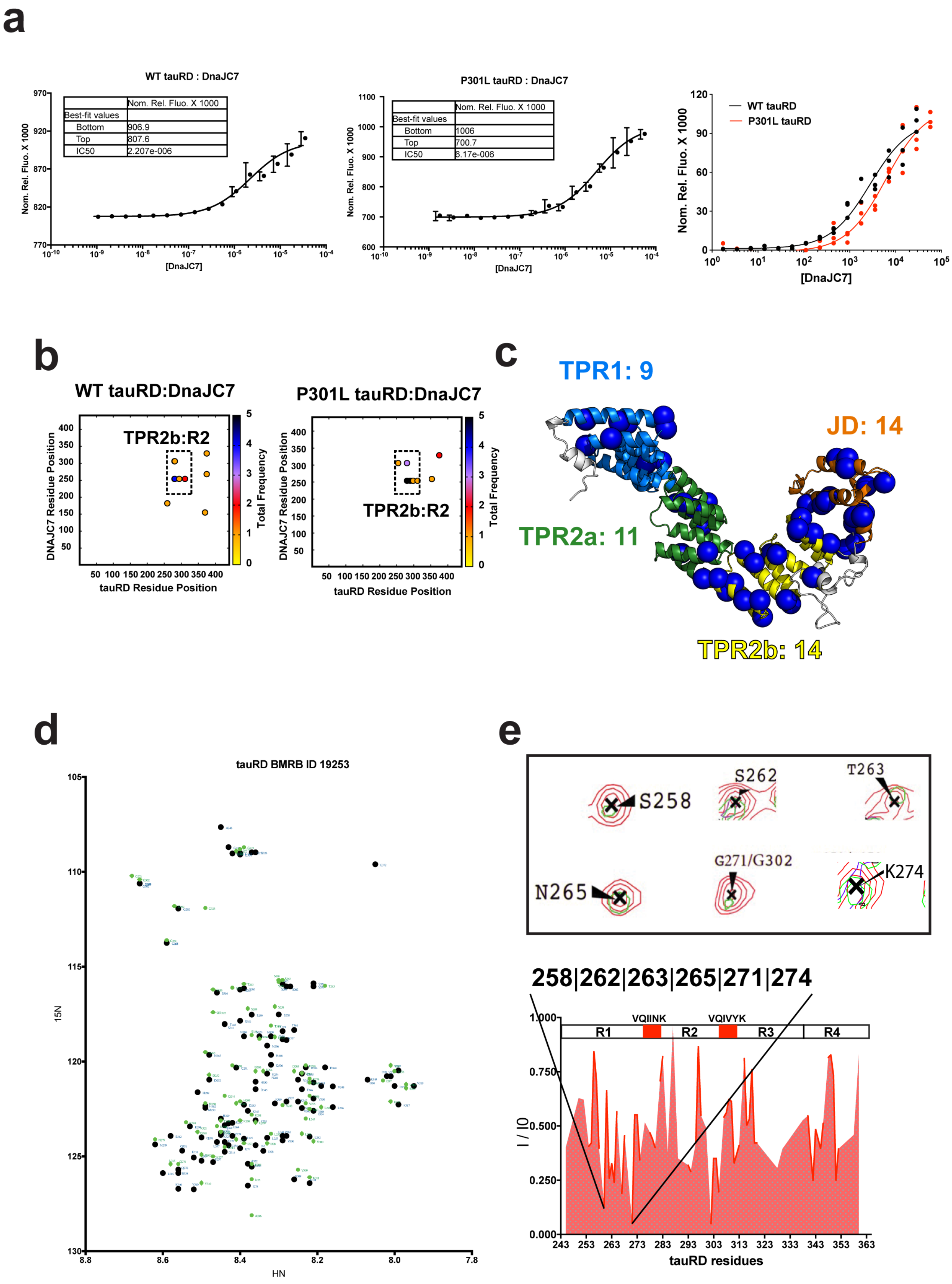

a
WT tauRD : DnaJC7
Nom. Rel. Fluo. X 1000
Best-fit values
Bottom 906.9
Top 807.6
IC50 2.207e-006
[DnaJC7]
P301L tauRD : DnaJC7
Nom. Rel. Fluo. X 1000
Best-fit values
Bottom 1006
Top 700.7
IC50 6.17e-006
[DnaJC7]
WT tauRD
P301L tauRD
Nom. Rel. Fluo. X 1000
[DnaJC7]
b
WT tauRD:DnaJC7
TPR2b:R2
DNAJC7 Residue Position
tauRD Residue Position
Total Frequency
P301L tauRD:DnaJC7
TPR2b:R2
DNAJC7 Residue Position
tauRD Residue Position
Total Frequency
c
TPR1: 9
JD: 14
TPR2a: 11
TPR2b: 14
d
tauRD BMRB ID 19253
15N
HN
e
S258
S262
T263
N265
G271/G302
K274
258|262|263|265|271|274
VQIINK
VQIVYK
R1
R2
R3
R4
I / I0
tauRD residues

Supplementary Figure 2. DnaJC7 binds to R1R2 in tauRD. **a**. Raw MST plots of DnaJC7 binding to WT tauRD (left panel) and P301L tauRD (middle panel). Overlay of normalized binding curves for WT tauRD:DnaJC7 (black) and P301L tauRD:DnaJC7 (red) showing the individual replicates (right panel). TauRD was labeled with Cyanine5 NHS ester dye (Cy5) and titrated by a serial two-fold dilution of DnaJC7. Data for each concentration is shown as a mean (n=3) with standard deviation. The data were fit to a linear regression model to estimate the binding constant. **b.** XL-MS to identify the contact maps between tauRD (WT and P301L) and DnaJC7. The recombinant WT and P301L tauRD were chemically crosslinked with DSS for one minute. After crosslinking, trypsin fragmentation and LC-MS analysis were performed. Each sample was carried out in five technical replicates. The crosslink pairs (in circles) are colored by average frequency across the replicates. **c.** Distribution of lysine residues on DnaJC7. DnaJC7 is colored as in Fig. 2d. Lysines are shown as spheres and are colored in blue. The frequency of lysines present in each domain is labeled. **d.** Overlay and amino acid assignments of submitted spectra of K18 245-372 (black, BMRB: 19253) and kind gift of K18 243-324 data (green) from Prof. Guy Lippens. **e**. Peak broadening from HSQC ^{15}N-^{1}H spectra comparing intensities from ^{15}N tauRD in the presence of 1X DnaJC7 (I) and 15N tauRD alone (I_o) reveals regions in the R1R2 element that broaden when DnaJC7 is bound. Inset shows peaks that broaden in the R1R2 region.

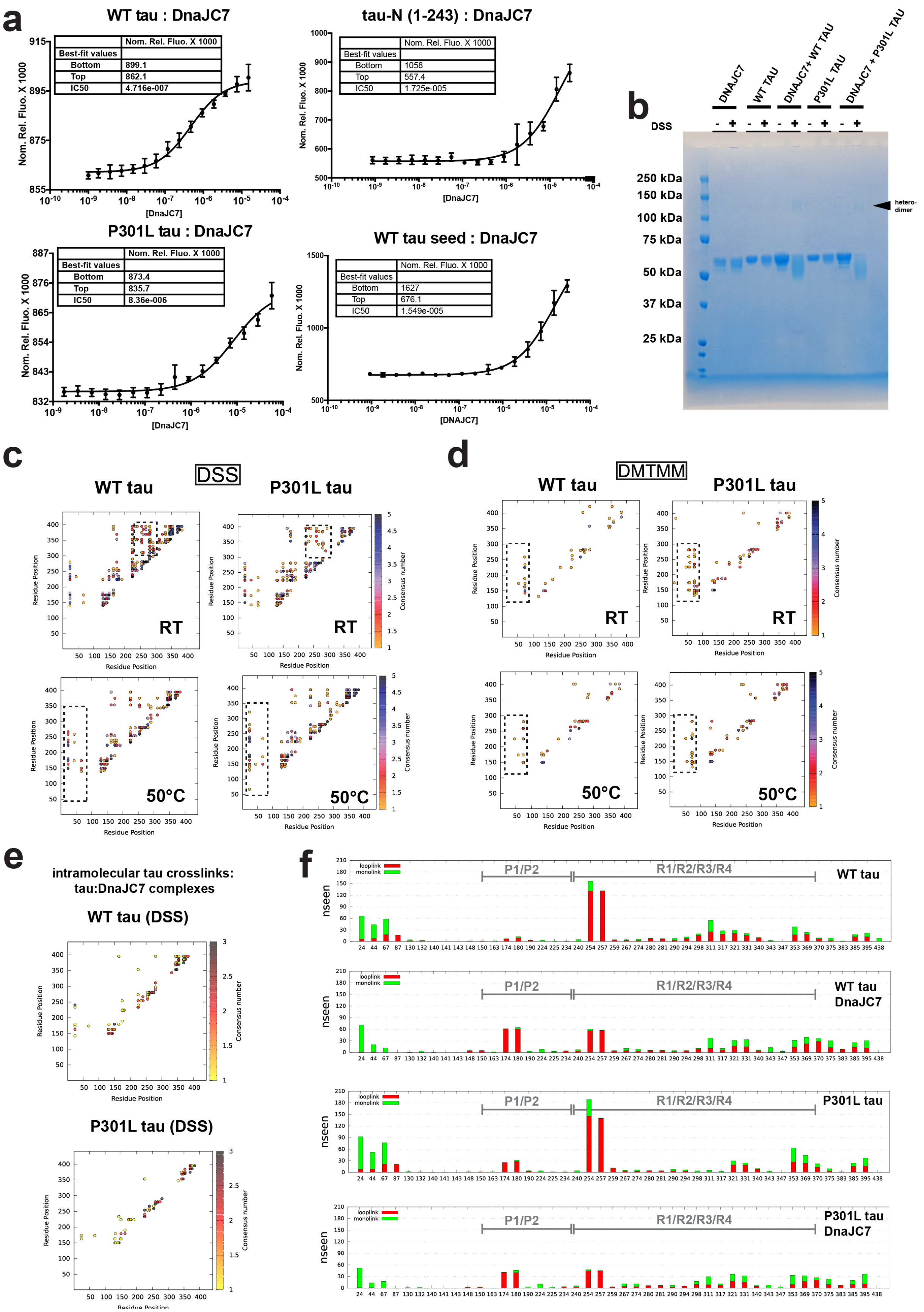

a
WT tau : DnaJC7
Nom. Rel. Fluo. X 1000
Best-fit values
Bottom 899.1
Top 862.1
IC50 4.716e-007
[DnaJC7]
tau-N (1-243) : DnaJC7
Nom. Rel. Fluo. X 1000
Best-fit values
Bottom 1058
Top 557.4
IC50 1.725e-005
[DnaJC7]
P301L tau : DnaJC7
Nom. Rel. Fluo. X 1000
Best-fit values
Bottom 873.4
Top 835.7
IC50 8.36e-006
[DnaJC7]
WT tau seed : DnaJC7
Nom. Rel. Fluo. X 1000
Best-fit values
Bottom 1627
Top 676.1
IC50 1.549e-005
[DNAJC7]
b
DSS
DNAJC7
WT TAU
DNAJC7+ WT TAU
P301L TAU
DNAJC7 + P301L TAU
250 kDa
150 kDa
100 kDa
75 kDa
50 kDa
37 kDa
25 kDa
hetero-dimer
c
DSS
WT tau
P301L tau
RT
50°C
Residue Position
Consensus number
d
DMTMM
WT tau
P301L tau
RT
50°C
Residue Position
Consensus number
e
intramolecular tau crosslinks: tau:DnaJC7 complexes
WT tau (DSS)
P301L tau (DSS)
Residue Position
Consensus number
f
looplink
monolink
nseen
P1/P2
R1/R2/R3/R4
WT tau
WT tau DnaJC7
P301L tau
P301L tau DnaJC7

Supplementary Figure 3. DnaJC7 prefers binding to natively folded tau. **a**. MST analysis of binding affinity between WT tau:DnaJC7, P301L tau:DnaJC7, N-term tau (1-243):DnaJC7 and tau seed:DnaJC7. WT tau, P301L tau, N-term tau and tau seed were labeled with Cyanine5 NHS ester dye (Cy5) and titrated by a serial two-fold dilution of DnaJC7. The experiments were performed as biological replicates (n=3) and each concentration point is shown as a mean with standard deviation. The data were fit to a linear regression model to estimate the binding constant. **b**. SDS-PAGE Coomassie gel of crosslinked WT tau:DnaJC7 and P301L tau:DnaJC7 complex with DSS. Only the bands containing DnaJC7 and tau were cut out for gel extraction followed by XL-MS analysis. The location of the hetero-dimer band is indicated by an arrow. SDS-PAGE of crosslinked samples was carried out at least 3 independent times. **c-d**. Consensus crosslink pairs of full-length WT/P301L tau (in circles) are shown in contact maps colored by average frequency across replicates using chemical reagent DSS (**c**) and DMTMM (**d**). In control, recombinant WT and P301L tau were heated at 50°C for one hour, then chemically crosslinked with DSS for one minute or DMTMM for 15 minutes. After crosslinking, gel extraction, trypsin fragmentation, and LC-MS analysis were performed. Each sample was carried out in five technical replicates. The dashed boxes emphasize the paired differences between WT and P301L tau. **e**. Consensus contact map of tau (WT, P301L) intramolecular crosslinks identified from the tau:DnaJC7 complexes colored by average frequency across replicates. **f.** Histogram of average frequencies of mono- and loop- crosslinks across replicates for WT tau in isolation and from the complex and P301L tau in isolation and from the complex. Monolinks and looplinks are in green and red, respectively. P1/P2 and repeat domain regions are highlighted with brackets.

a

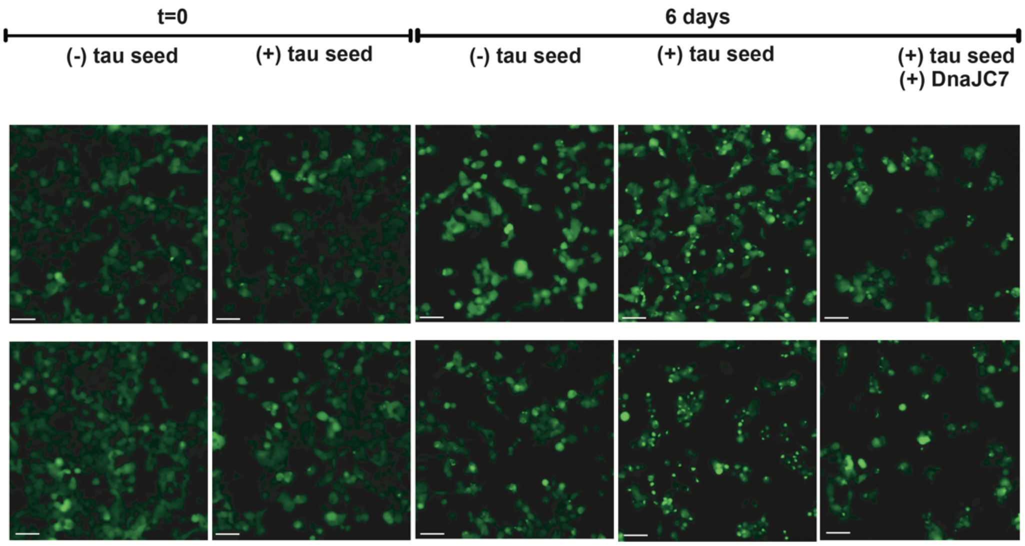

b

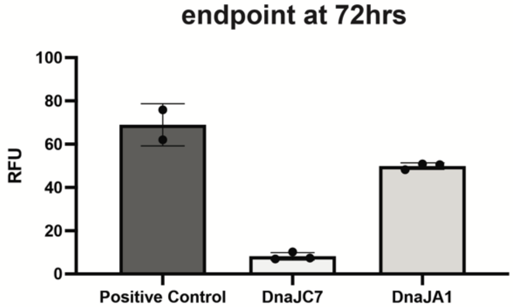

Supplementary Figure 4. DnaJC7 suppression of tau aggregation does not produce seeds. **a**. Seeding activity in tau RD-CFP/YFP biosensor cells. Full-length tau (WT and P301L) incubated with M_s or DnaJC7 from previous ThT experiments were seeded into cells via lipofectamine transfection (Methods) at Day 0 (T=0 days) and Day 6 (T=6 days). At T0, WT and P301L tau only show background FRET signal from M_s. At T=6 days, both WT and P301L exhibit strong seeding activity in condition with M_s but decreased activity in condition with M_s and DnaJC7. All seeding experiments were carried out as biological triplicates. The white bar represents 20 μm distance in each image. **b.** Specificity between DnaJC7 and canonical DnaJ family member proteins attributed to tau anti-aggregation effects. After 72 hours incubation, tau incubated with different DnaJ proteins were transduced into biosensor cells. FRET (tau RD-mClover/mCerulean) from each condition was measured by flow cytometry as biological replicates (n=3) and shown as a mean with standard deviation.

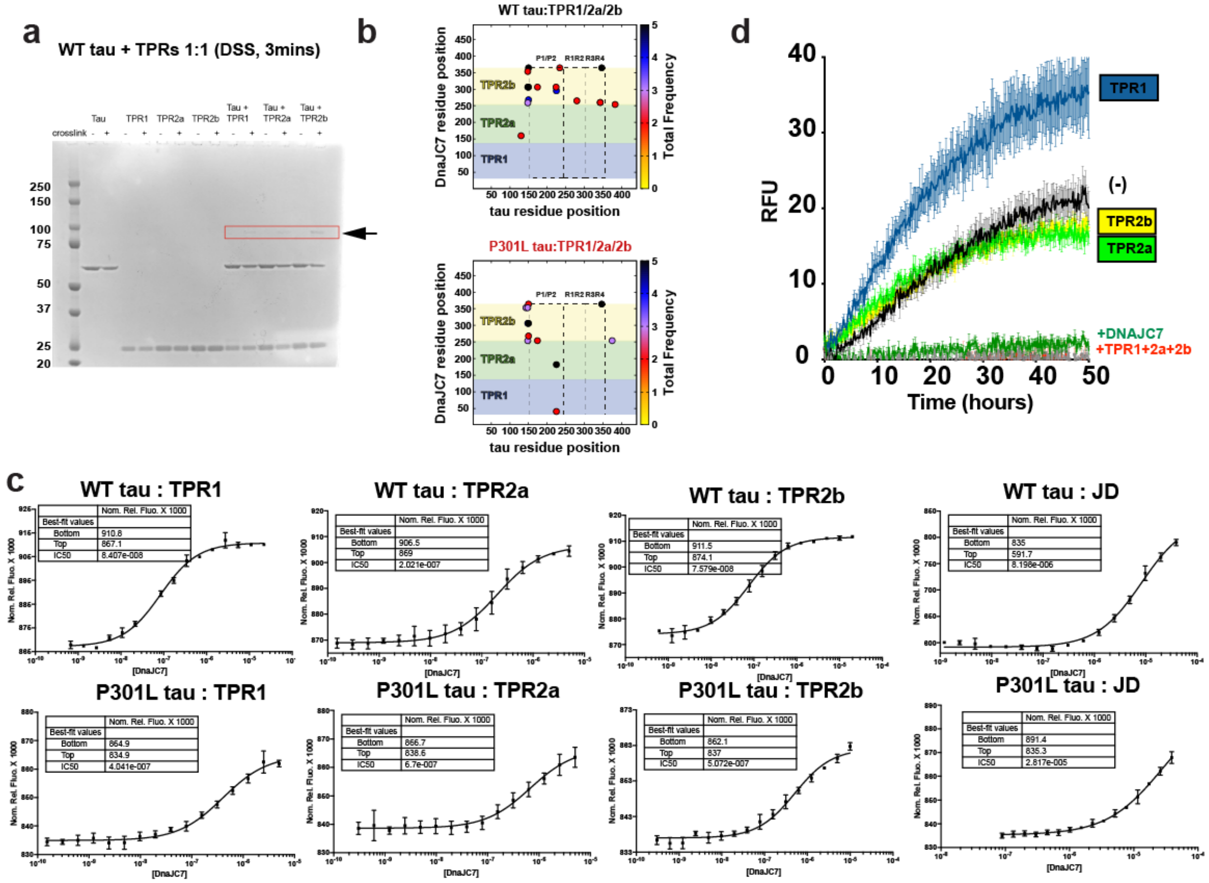

Supplementary Figure 5. DnaJC7's anti-aggregation activity relies on all three TPR domains. a. SDS-PAGE Coomassie gel of crosslinked TPR domain:tau complex. Recombinant TPR domains and full-length WT tau were incubated at RT for one hour then chemically cross-linked using DSS. Only the bands forming complex (red mark) are cut out for gel extraction followed by XL-MS analysis. The hetero-dimer band is indicated by an arrow. SDS-PAGE of crosslinked samples was carried out at least 3 independent times. **b**. Overlay contact map of DSS crosslinks identified for each single TPR domain:WT tau to full-length WT tau:DnaJC7. The crosslink pairs are colored by domain TPR1 (yellow), TPR2a (red), TPR2b (purple) and full-length DnaJC7 (black). The y-axis is colored according to DnaJC7 domain. The TPR2b: WT tau crosslink pairs recapitulate the contacts observed in full-length DnaJC7. **c.** MST analysis of affinity between WT tau with DnaJC7 domains (upper panel) and P301L tau with DnaJC7 domains (lower panel). WT tau and P301L tau were labeled with Cyanine5 NHS ester dye (Cy5) and titrated by a serial two-fold dilution of DnaJC7 domains. The binding experiments were performed as biological replicates (n=3) and are shown as mean with standard deviation. **d**. ThT experiment that shows that a single TPR domain is not sufficient to depress tau aggregation. Full-length DnaJC7 or combination of three TPRs efficiently depress the tau aggregation. 4.4 µM P301L tau aggregation was induced using 50 nM tau seeds in the presence of different DnaJC7 TPR domains. Aggregation was monitored via ThT fluorescence. Experiments were performed as biological replicates (n=3) and are shown as a mean with standard deviation. Individual DnaJC7 domains were added in an equimolar ratio to tau.

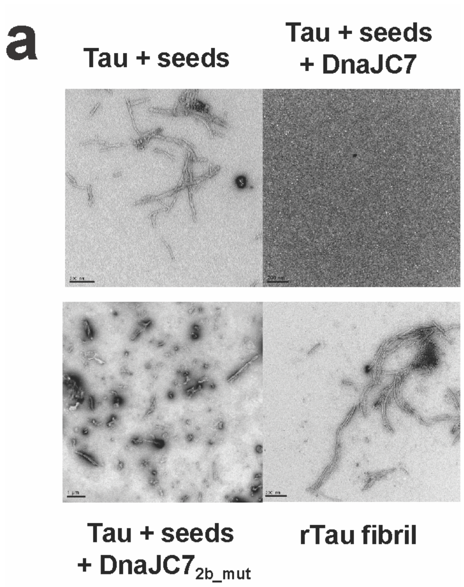

Supplementary Figure 6. DnaJC7$_{TPR2b_mut}$ is unable to suppress tau aggregation efficiently. a. TEM images of endpoint ThT fluorescence tau aggregation experiments. Fibrils were detected in tau seeded reactions and comparable to recombinant tau fibrils positive samples. Presence of equimolar concentrations of DnaJC7$_{WT}$ inhibits formation of fibrils but DnaJC7$_{TPR2b_mut}$ does not. TEM of the aggregated samples was carried out at least 3 independent times. Scale bar for each micrograph is 200 nm.

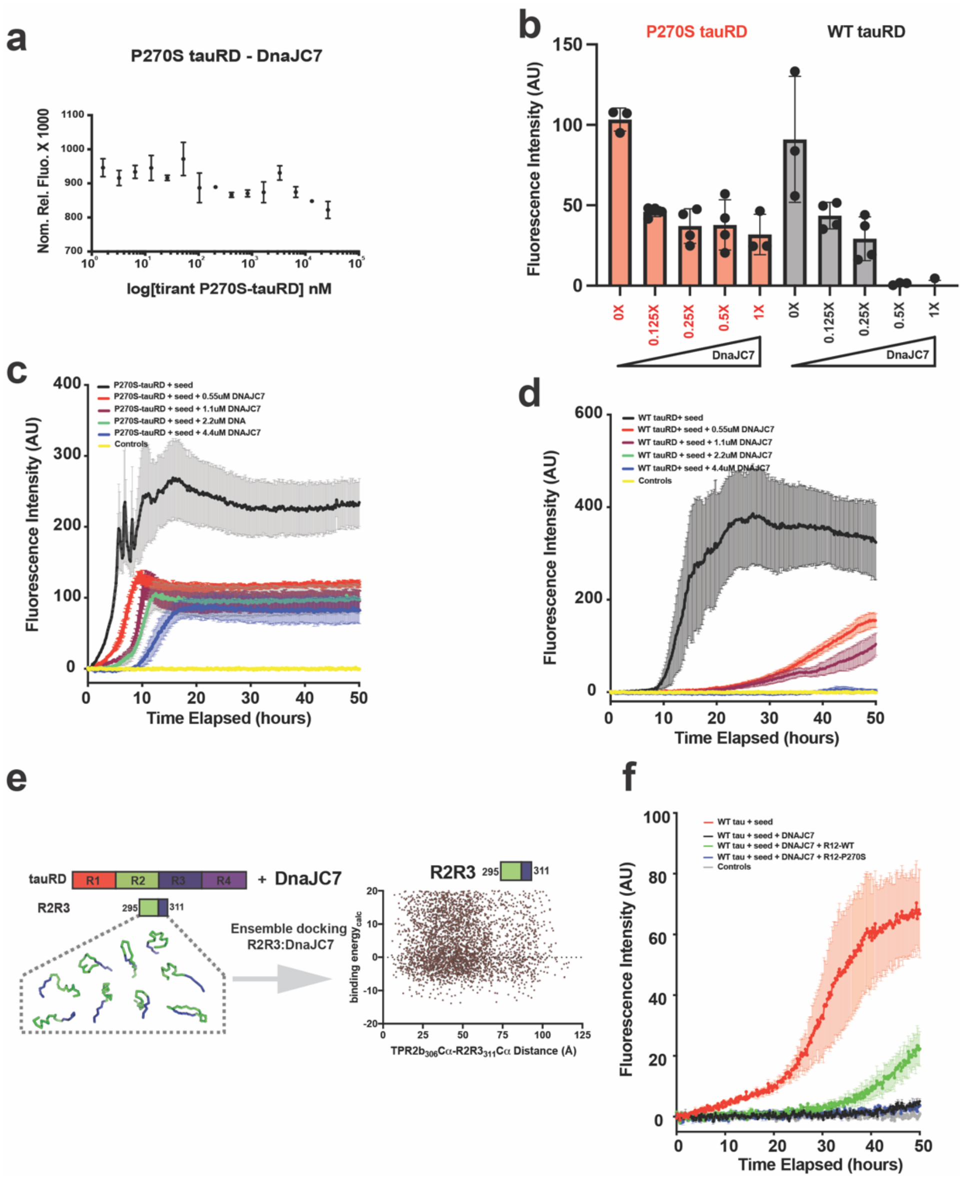

Supplementary Figure 7. P270S mutation in R1R2 prevent recognition by DnaJC7. **a.** MST analysis shows no binding affinity between P270S tau RD with DnaJC7. DnaJC7 was labeled with Cyanine5 NHS ester dye (Cy5) and titrated by a serial two-fold dilution of P270S tauRD. The experiments were done as technical replicates (n=3) and are shown as a mean with standard deviation. **b**. Bar plot of end point values (at 50 hours) for seeded aggregation kinetics of 4.4μM P270S tauRD (red) and 4.4μM WT tauRD (black) in the presence of 0X, 0.125X, 0.25X, 0.5X and 1X DnaJC7 concentrations. (**c-d**) Raw ThT aggregation traces of P270S tauRD and WT tauRD in the presence of increasing concentrations of DnaJC7 over 50 hours. ThT signals are shown as a mean (n=3) with standard deviation. **e.** Schematic illustrating the generation of an ensemble of R2R3 peptide conformations used in the DnaJC7 docking simulation. The R2R3 peptides are shown in cartoon representation and colored green/blue according to the repeat domain. Calculated binding energy and crosslink geometry for

the R2R3:DnaJC7 structural ensemble. Each point represents a structural model. Models with low binding energies and short Cα-Cα distances between K306 (DnaJC7) and K311 (tau) were used in subsequent analyses. **f.** ThT aggregation reactions of 4.4μM WT tau, 50nM tau monomer seeds and 4.4μM DnaJC7 in the presence of buffer (black), P270S peptide R1R2 (blue), WT peptide R1R2 (green). Control 4.4μM WT tau with 50nM tau monomer seeds is shown in red. Individual component controls are shown in grey. ThT signals are shown as a mean (n=3) with standard deviation.
