# Supplementary material for: DnaJC7 binds natively folded structural elements in tau to inhibit amyloid formation
Source: Nat Commun. 2021 Sep 9;12:5338. doi: 10.1038/s41467-021-25635-y (PMC8429438; doi:10.1038/s41467-021-25635-y)
Supplement: Supplementary file 3 — Reporting Summary [file 41467_2021_25635_MOESM3_ESM.pdf]

nature research

Corresponding author(s): LA Joachimiak
Last updated by author(s): Aug 20, 2021

Reporting Summary

Nature Research wishes to improve the reproducibility of the work that we publish. This form provides structure for consistency and transparency in reporting. For further information on Nature Research policies, see Authors & Referees and the Editorial Policy Checklist.

Statistics

For all statistical analyses, confirm that the following items are present in the figure legend, table legend, main text, or Methods section.

n/a	Confirmed	
☐	☒	The exact sample size (*n*) for each experimental group/condition, given as a discrete number and unit of measurement
☐	☒	A statement on whether measurements were taken from distinct samples or whether the same sample was measured repeatedly
☒	☐	The statistical test(s) used AND whether they are one- or two-sided<br>*Only common tests should be described solely by name; describe more complex techniques in the Methods section.*
☒	☐	A description of all covariates tested
☒	☐	A description of any assumptions or corrections, such as tests of normality and adjustment for multiple comparisons
☐	☒	A full description of the statistical parameters including central tendency (e.g. means) or other basic estimates (e.g. regression coefficient) AND variation (e.g. standard deviation) or associated estimates of uncertainty (e.g. confidence intervals)
☒	☐	For null hypothesis testing, the test statistic (e.g. *F*, *t*, *r*) with confidence intervals, effect sizes, degrees of freedom and *P* value noted<br>*Give P values as exact values whenever suitable.*
☒	☐	For Bayesian analysis, information on the choice of priors and Markov chain Monte Carlo settings
☒	☐	For hierarchical and complex designs, identification of the appropriate level for tests and full reporting of outcomes
☒	☐	Estimates of effect sizes (e.g. Cohen's *d*, Pearson's *r*), indicating how they were calculated

Our web collection on statistics for biologists contains articles on many of the points above.

Software and code

Policy information about availability of computer code

Data collection	All DNAJC7 and tauRD protein simulations were carried out with ROSETTA (available at https://www.rosettacommons.org/). NMR data was acquired using Bruker Topspin v3.5 (available at https://www.bruker.com/service/support-upgrades/software-downloads/nmr.html). MST data was acquired by NanoTemper Microscale Thermophoresis (available at https://nanotempertech.com/monolith/). Conservation was calculated using Al2Co (available at http://prodata.swmed.edu/download/pub/al2co/). FRET data was collected using BD LSRFortessa (available at https://www.bdbiosciences.com/en-in/instruments/research-instruments/research-cell-analyzers/lsrfortessa).
Data analysis	All images of structures were produced in pymol v1.8.4.2. All plots were generated with GraphPad Prism 8 and gnuplot. Mass spectrometry data were analyzed using Proteome Discoverer v2.4 (available at https://www.thermofisher.com/us/en/home/industrial/mass-spectrometry/liquid-chromatography-mass-spectrometry-lc-ms/lc-ms-software/multi-omics-data-analysis/proteome-discoverer-software.html). XL-MS data analysis was carried out using Xquest v2.1.3 (available at http://proteomics.ethz.ch/cgi-bin/xquest2_cgi/index.cgi). Solution NMR data was processed using NMRpipe v10.9 (available https://www.ibbr.umd.edu/nmrpipe/install.html) and analyzed SPARKY (available https://www.cgl.ucsf.edu/home/sparky/). MST data was analyzed using PALMIST v1.5.1 (available at https://www.utsouthwestern.edu/labs/mbr/software/). FRET data was analyzed using FlowJo v10 (available at https://www.flowjo.com/solutions/flowjo/downloads).

For manuscripts utilizing custom algorithms or software that are central to the research but not yet described in published literature, software must be made available to editors/reviewers. We strongly encourage code deposition in a community repository (e.g. GitHub). See the Nature Research guidelines for submitting code & software for further information.

Data

Policy information about availability of data

All manuscripts must include a data availability statement. This statement should provide the following information, where applicable:

- Accession codes, unique identifiers, or web links for publicly available datasets
- A list of figures that have associated raw data
- A description of any restrictions on data availability

Raw mass spectrometry data for tau immunoprecipitation is available in Source Data 1. Raw crosslinking mass spectrometry data is available in Source Data 2. Raw data for all for western blots, FRET tau biosensor analysis, MST binding measurements, NMR chemical shift analysis, tauRD Rosetta simulations, ThT fluorescence aggregation experiments, and Rosetta docking simulations is available in Source Data 3. Any other supporting data is available upon reasonable request from the authors.

Field-specific reporting

Please select the one below that is the best fit for your research. If you are not sure, read the appropriate sections before making your selection.

☒ Life sciences ☐ Behavioural & social sciences ☐ Ecological, evolutionary & environmental sciences

For a reference copy of the document with all sections, see nature.com/documents/nr-reporting-summary-flat.pdf

Life sciences study design

All studies must disclose on these points even when the disclosure is negative.

Sample size	We did not study populations either of animals or humans, thus population sample size is not applicable. For the PS19 mouse immunoprecipitation experiments, each time point (weeks 1, 2, 3, 4, 5 and 6) included three mouse brains (n=3). In the case of Rosetta simulations, we chose 5000 as the number of models to build for the tau repeat domain modeling experiments. For the ensemble docking experiments we built 5000 low resolution models, each model was then refined using the full atom energy function. Using the biohpc computer cluster at UTSW this represented 2 weeks of computer time. These sample sizes are relatively standard in the field considering the size of the protein (Weitzner et al. Nat Protocol 2017). For the FRET tau biosensor, in vitro aggregation and binding experiments no sample size calculation was performed. These experiments were performed in triplicate to demonstrate reproducibility of each assay.
Data exclusions	No data were excluded in the analyses
Replication	Figure 1 and Supplementary Figure 1. Mass spectrometry analysis of tau IP from PS19 tauopathy mice (probed with HJ8.5 anti-human tau antibody) across different ages was performed once. The mass spectrometry data were analyzed using Proteome Discoverer (Thermo) and raw data was available in Source Data 1. The evaluation of pathogenic seeds in cells was carried out in P310S FRET biosensor cells (ATCC, CRL-3275). The cells were analyzed by FACS and the data analyzed using FloJo in triplicate on at least 10,000 cells from populations of 30,000 cells, reported as averages with standard deviation. Western blot analysis of DNAJC7 Immunoprecipitation (IP) was performed one time using anti-DNAJC7 antibody (Proteintech, 11090-1-AP). Dose titration of DNAJC7 KO cell line was carried out in as triplicates on at least 10,000 from populations of 100,000 cells and shown as averages with standard deviation.<br>Figure 2 and Supplementary Figure2. MST assay was performed three independent times with 16 dose points and are shown as averages with standard deviation. Crosslink experiments were performed five independent times. Raw XL-MS data is reported in Source Data 2.<br>1000 homology models of DNAJC7 were built with ROSETTA using the P58 (IPK) as a template (PDB ID: 3ieg), a representative model from low energy criteria is shown to highlight the residues matching the XL-MS data. Solution HSQC NMR experiments on 15N TauRD were performed once.<br>Figure 3 and Supplementary Figure 3. MST assay was performed three independent times with 16 dose points and are shown as averages with standard deviation. Crosslink experiments were performed five independent times. Raw XL-MS data is reported in Source Data 2.<br>Figure 4 and Supplementary Figure 4. ThT aggregation assay was performed in triplicates and data was plotted as averages with standard deviation. TEM was performed once. FACS analysis of P310S FRET biosensor cells (ATCC, CRL-3275) was performed as three biological replicates. Quantification of frequency of puncta was performed using FLoJo in triplicate on at least 10,000 cells from populations of 20,000 cells, reported as averages with standard deviation.<br>Figure 5 and Supplementary Figure 5. MST assay was performed three independent times with 16 dose points and are shown as averages with standard deviation. The ThT aggregation assay was performed as technical triplicates and data was plotted as averages with standard deviation.The SDS-PAGE gel was carried out once. Crosslink experiments were performed three independent times. Raw XL-MS data is reported in Source Data 2.<br>Figure 6 and Supplementary Figure 6. MST assay was performed three independent times with 16 dose points and are shown as averages with standard deviation. The ThT aggregation assay was performed as technical triplicates and data was plotted as averages with standard deviation. FACS analysis of P310S FRET biosensor cells (ATCC, CRL-3275) was performed as three biological replicates. Quantification of frequency of puncta was performed using FLoJo in triplicate on at least 10,000 cells from populations of 20,000 cells, reported as averages with standard deviation. TEM was performed once.<br>Figure 7 and Supplementary Figure 7. 5000 model ensemble of tau repeat domain were built with CS-ROSETTA guided by backbone chemical shift assignments from bmrb (https://bmrb.io/data_library/summary/index.php?bmrbId=19253) and also kindly provided by Guy Lippens. Low energy conformation and Ca-Ca distance restriction from XL-MS data were used to produce the DNAJC7:R1R2 complex in UTSW biohpc cluster. The ThT aggregation assay was performed in triplicates and data was plotted as averages with standard deviation.<br>Figure 8. N/A

Randomization	Samples were not allocated into groups, so randomization is not applicable.
Blinding	Samples were not allocated into groups; blinding was not applied.

Reporting for specific materials, systems and methods

We require information from authors about some types of materials, experimental systems and methods used in many studies. Here, indicate whether each material, system or method listed is relevant to your study. If you are not sure if a list item applies to your research, read the appropriate section before selecting a response.

Materials & experimental systems

n/a	Involved in the study
☐	☒ Antibodies
☐	☒ Eukaryotic cell lines
☒	☐ Palaeontology
☐	☒ Animals and other organisms
☒	☐ Human research participants
☒	☐ Clinical data

Methods

n/a	Involved in the study
☒	☐ ChIP-seq
☐	☒ Flow cytometry
☒	☐ MRI-based neuroimaging

Antibodies

Antibodies used	Mouse anti-MAPT Recombinant Antibody (clone HJ8.5) (CAT#: NS-090CN)<br>Rabbit anti-MAPT recombinant antibody (Agilent, A002401-2)<br>rabbit IgG control (Abcam, ab37415)<br>Rabbit anti-DnaJC7 antibody (Proteintech, 11090-1-AP)<br>Veriblot HRP (Abcam, ab131366)<br>Donkey anti-rabbit-HRP (Cytiva, NA9340-1ML)
Validation	Antibodies on this study were validated by the vendor to specifically recognize the target

Eukaryotic cell lines

Policy information about cell lines

Cell line source(s)	Tau RD P301S FRET Biosensor (ATCC CRL-3275)<br>293T/17 [HEK293T/17] (ATCC CRL-1268)
Authentication	Cell lines were not authenticated
Mycoplasma contamination	Cells were confirmed to be free of mycoplasma contamination
Commonly misidentified lines (See ICLAC register)	No misidentified cell lines were used in this study
